# CT characteristics and their clinicopathological associations in ground-glass nodular multifocal lung adenocarcinoma

**DOI:** 10.1097/MD.0000000000042145

**Published:** 2025-04-25

**Authors:** Meng Zhou, Jiuchen Wang, Huadong Chen, Weihua Tang, Chunlin Li

**Affiliations:** a Radiology Department, The Central Hospital of Enshi Tujia and Miao Autonomous Prefecture, Enshi, Hubei, China; b Department of Minimally Intervention Therapy, Cancer Hospital of Shantou University Medical College, Shantou, Guangdong, China.

**Keywords:** clinicopathological characteristics, CT, diagnosis, multifocal lung adenocarcinoma with ground-glass nodules

## Abstract

This study investigates the correlation between CT findings and clinicopathological features in patients with ground-glass nodular multifocal lung adenocarcinoma. A total of 203 lesions were identified and classified based on pathological results into 3 groups: preinvasive lesions (92 nodules), microinvasive adenocarcinoma (69 nodules), and invasive adenocarcinoma (42 nodules). CT imaging was performed on all patients, and the CT features were analyzed in relation to clinicopathological characteristics. Statistical analysis was performed using SPSS 22.0 software. Continuous variables were compared using one-way ANOVA or the Kruskal–Wallis test, while categorical variables were analyzed using the Chi-square test or Fisher exact test. Spearman correlation analysis was used to assess the relationship between CT features and clinicopathological characteristics. Significant differences were observed in nodule morphology, lobulation, vacuolation, burr sign, air bronchial sign, pleural indentation, tumor–lung interface, vascular characteristics, and ground-glass nodule type between the 3 groups (*P* < .05). The invasive adenocarcinoma group exhibited higher proportions of round/oval nodules, lobulation, vacuolation, burr sign, air bronchial sign, pleural depression, clear tumor–lung interface, vascular penetration, and partial solid nodules compared to the other 2 groups (*P* < .05). The microinvasive adenocarcinoma group showed a higher incidence of vacuolation, burr sign, pleural indentation, vascular penetration, and partial solid nodules than the preinvasive group (*P* < .05). No significant differences were found in the nodule locations among the groups (*P* > .05). The average size of nodules in the invasive group was significantly larger than in the other 2 groups (*P* < .05), while no difference was observed between the preinvasive and microinvasive groups (*P* > .05). The incidence of several CT signs, such as burr sign and pleural depression, negatively correlated with tumor differentiation (*r* = −0.311 to −0.378, all *P* < .05). The occurrence of pure ground-glass nodules was positively correlated with differentiation (*R* = 0.127, *P* < .05). Additionally, lesion characteristics like shape and lobulation were linked to lymph node metastasis (*R* = 0.313 to 0.415, *P* < .05). CT features in multifocal lung adenocarcinoma patients are closely related to pathological characteristics, providing valuable insights for clinical diagnosis and classification.

## 1. Introduction

With the popularization of lung cancer screening programs, the detection rate of ground-glass nodules (GGNs) in lung has increased significantly. Ground-glass pulmonary nodules are a kind of nodules with a high degree of malignancy, which is closely related to lung cancer.^[1]^ Lung adenocarcinoma is the most common histological type of lung cancer. According to the degree of invasion, it can be divided into preinvasive lesions (atypical adenomatous hyperplasia and adenocarcinoma in situ), microinvasive adenocarcinoma, and invasive adenocarcinoma, which may be shown as GGNs in CT. How to accurately diagnose and judge the invasion and metastasis of lung adenocarcinoma? It is of great significance for the formulation of clinical treatment plan, namely prognosis assessment.^[2]^

Although previous studies have extensively investigated the CT features of solitary GGNs, research on multifocal lung adenocarcinoma (MLA) with GGNs remains limited. Most existing studies focus on single nodules, which may not fully capture the complexity and heterogeneity of MLA.^[3–6]^ Furthermore, the correlation between CT findings and clinicopathological characteristics in MLA patients has not been thoroughly explored. For instance, the diagnostic value of specific CT signs, such as vacuolation, lobulation, and vascular changes, in differentiating preinvasive, microinvasive, and invasive lesions remains unclear. Additionally, the relationship between CT features and lymph node metastasis in MLA patients has not been systematically studied, leaving a gap in the understanding of how imaging characteristics can predict tumor behavior and guide treatment decisions.

Therefore, the purpose of this study is to investigate the relationship between CT findings and clinicopathological features of ground-glass nodular MLA, and to provide reference for clinical diagnosis and treatment. The details are reported as follows.

## 2. Objects and methods

### 2.1. Subjects

This study was approved by the Ethics Committee of the Central Hospital of Enshi Tujia and Miao Autonomous Prefecture. All data collected in this study were obtained with the informed consent of the patients from the Cancer Hospital of Shantou University Medical College. A total of 80 patients with MLA treated in our hospital from July 2018 to August 2021 were included as subjects.

Inclusion criteria: (1) all patients underwent CT examination, and the imaging findings indicated multiple GGNs in the lung. (2) MLA was confirmed by pathological examination. (3) The maximum diameter of the lesion was within 3 cm. (4) Clinical data were complete.

Exclusion criteria: (1) patients who had received antitumor therapy before the examination. (2) Patients with pulmonary nodules affected by severe underlying lung diseases, such as severe emphysema or pulmonary fibrosis, which could interfere with the accurate assessment of nodule characteristics. (3) CT images with significant artifacts that could affect observation and measurement.

To ensure the representativeness of the sample, we rigorously screened all potential cases during the study period. Only patients who met the inclusion criteria and had no exclusion factors were enrolled. This approach minimized selection bias and ensured that the study population consisted of typical cases of MLA. Among the 80 patients, 28 were male and 52 were female, with an average age of 52.69 ± 7.93 years (range: 32–70 years). The distribution of nodules was as follows: 47 cases had 2 nodules, 23 cases had 3 nodules, and 10 cases had 4 nodules.

### 2.2. CT examination method

GE Light Speed VCT 64-slice spiral CT was used, with the patient in supine position, arms raised, head advanced. The parameters of conventional chest CT scanning were tube voltage of 120 kV, tube current of 150 mA, layer thickness of 5 mm, layer spacing of 5 mm, pitch of 0.984:1. The patient was instructed to hold breath after inhalation before scanning, and the scanning range was from the tip of the lung to below the diaphragm and included all lung tissues. The target scanning parameters are tube voltage 140 kV, tube current 300 mA, layer thickness 0.625 mm, layer spacing 0.625 mm, matrix 512 × 512, pitch 0.984:1, FOV: 20 to 25 cm. The images obtained from the scan were sent to the pACS system through the workstation and observed in the pulmonary window and mediastinal window. The lesions were observed by multiplanar reconstruction, maximum density projection, minimum density projection and other methods, and the images were read by 2 diagnostic imaging physicians in a blind way. The structural features of nodules were observed, including: nodal position (left upper lobe, left lower lobe, the upper leaves, middle of the right lung, right lung lower lobe), nodular morphology (irregular, round/oval), edge character (burr), Ye Zheng, pleural sag), internal features, air bronchogram, cavitation), vascular characteristics (through changes in blood vessels, blood vessels), and the size of the nodule (nodules coronal, sagittal, transverse maximum size the mean). GGNs are divided into partial solid nodules and pure GGNs according to whether they contain parenchymal components.

### 2.3. Pathological diagnosis

The specimens obtained by surgery were fixed and embedded, and serial sections were stained with hematoxylin and eosin staining. Preinvasive lesions: focal lesions <3 cm in diameter with tumor growth along the alveolar wall without interstitial or vascular infiltration. Microinvasive adenocarcinoma: focal lesions <3 cm in diameter, mainly adherent growth of tumor cells, infiltration of <0.5 cm; invasive adenocarcinoma: a focal lesion <3 cm in diameter with an invasion of > 0.5 cm. According to the pathological results, the patients were divided into preinvasive lesion group (92 nodules), microinvasive adenocarcinoma group (69 nodules), and invasive adenocarcinoma group (42 nodules).

### 2.4. Observation indicators

CT signs and clinicopathological features of the 3 groups were analyzed.

### 2.5. Statistical analysis

SPSS 22.0 software was used. The measurement data were described as mean ± standard deviation ($\bar{x}\pm s$) in accordance with normal distribution, and the independent *t* test was used for comparison between the 2 groups. Enumeration data were expressed as N (%), *χ*^2^ test was used for comparison between groups, and *t* test was used for comparison of rank data. Spearman rank correlation coefficient was used for correlation analysis. *P* < .05 was considered statistically significant.

## 3. Results

### 3.1. Comparison of CT global signs among the 3 groups

There were significant differences in nodule morphology, lobulation sign, vacuolation sign, burr sign, air bronchial sign, pleural indentation sign, tumor–lung interface, vascular characteristics, and GGN type among the preinvasive lesion group, microinvasive adenocarcinoma group, and invasive adenocarcinoma group (*P* < .05). The proportion of round/oval nodules, lobulated sign, vacuole sign, burr sign, air bronchial sign, pleural depression sign, clear tumor–lung interface, vascular penetration, and partial solid nodules in the invasive adenocarcinoma group were higher than those in the other 2 groups (*P* < .05). The proportion of vacuolar sign, burr sign, pleural depression sign, vascular penetration, and partial solid nodules in the microinvasive adenocarcinoma group were higher than those in the preinvasive lesion group (*P* < .05), and there was no significant difference in the location distribution of nodules among the 3 groups (*P* > .05), as shown in Table 1. *P*-values were calculated using the Chi-square test for categorical variables.

**Table 1:** CT signs in different groups of pulmonary nodules.

CT signs	Preinvasive lesion group (92 nodules)	Microinvasive adenocarcinoma group (69 nodules)	Invasive adenocarcinoma group (42 nodules)	χ² value	*P*-value
Location					
Upper right	28 (30.43%)	17 (24.64%)	15 (35.71%)	2.960	.923
Right lower	15 (16.30%)	13 (18.84%)	4 (9.52%)		
Left upper	17 (18.48%)	13 (18.84%)	9 (21.43%)		
Left lower	15 (16.30%)	12 (17.39%)	6 (14.29%)		
Form					
Round/oval	69 (75.00%)	47 (68.12%)	14 (33.33%)*	22.49	<.001
Irregular	23 (25.00%)	22 (31.88%)	28 (66.67%)		
Lobulation					
With lobulation	37 (40.22%)	32 (46.38%)	28 (66.67%)	8.168	.017
Without lobulation	55 (59.78%)	37 (53.62%)	14 (33.33%)		
Cavitation					
With cavitation	23 (25.00%)	30 (43.48%)	18 (42.86%)	7.366	.025
Without cavitation	69 (75.00%)	39 (56.52%)	24 (57.14%)		
Burr sign					
With burr sign	47 (51.09%)	52 (75.39%)	34 (80.95%)	15.86	<.001
Without burr sign	45 (48.91%)	17 (24.61%)	8 (19.05%)		
Air bronchial sign					
With air bronchial sign	13 (14.13%)	17 (24.64%)	24 (57.14%)	27.53	<.001
Without air bronchial sign	79 (86.87%)	52 (75.36%)	18 (42.86%)		
Pleural depression sign					
With pleural depression sign	23 (25.00%)	40 (57.97%)	34 (80.95%)	40.530	<.001
Without pleural depression sign	69 (75.00%)	29 (42.03%)	8 (19.05%)		
Tumor–lung interface					
Clear Interface	45 (48.91%)	36 (52.17%)	34 (80.95%)	12.906	.002
Fuzzy interface	47 (51.09%)	33 (47.83%)	8 (19.05%)		
Vascular characteristics					
Vascular changes	64 (69.57%)	22 (31.88%)	7 (16.67%)	40.673	<.001
Through blood vessels	28 (30.43%)	47 (68.12%)	35 (83.33%)		
Ground glass nodule type					
Pure ground glass nodules	83 (90.22%)	35 (50.72%)	0 (0.00%)	98.782	<.001
Partial solid nodules	9 (9.78%)	34 (39.28%)	42 (100.00%)		

* Compared with the preinvasive lesion group, *P* < .05.

### 3.2. Comparison of CT quantitative parameters among the 3 groups

Nodules on average size, average size solid nodules before infiltration lesion group, the micro infiltrating adenocarcinoma group, the differences between the infiltrating adenocarcinoma groups was statistically significant (*P* < .05), 2 further comparison, infiltrating adenocarcinoma were greater than the other 2 groups (*P* < .05), lesion before infiltration and micro infiltrating adenocarcinoma group had no difference between statistical significance (*P* > .05), are shown in Table 2. *P*-values were calculated using the *t* test for groups.

**Table 2:** Comparison of CT quantitative parameters among the 3 groups (^*x*^ ± s).

Group	Mean nodule size (mm)	Mean size of solid nodules (mm)
Preinvasive lesion group (92 nodules)	10.74 ± 4.72	3.50 ± 1.24
Microinvasive	11.85 ± 5.15	3.89 ± 1.72
Adenocarcinoma group (69 nodules)		
Invasive	19.07±3.24*†	8.75±1.47*†
Adenocarcinoma group (42 nodules)		
*F*	49.363	201.677
*P*	<.001	<.001

* Compared with the preinvasive lesion group, *P* < .05.

† Compared with the microinvasive adenocarcinoma group, *P* < .05.

### 3.3. Comparison of CT images and pathological findings

**Figure 1.**
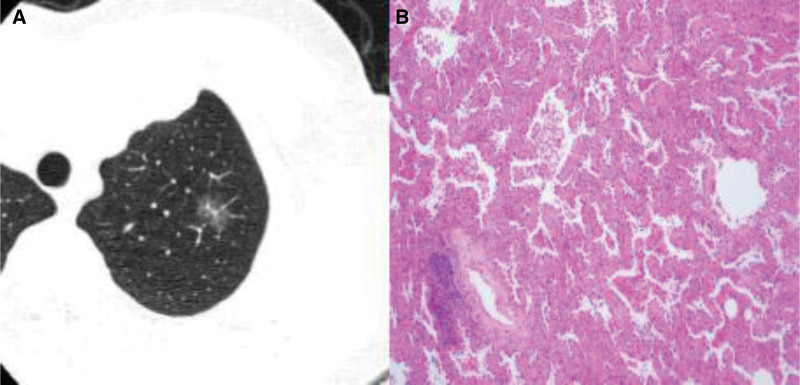
(A) The CT image and (B) the pathological image.

**Figure 2.**
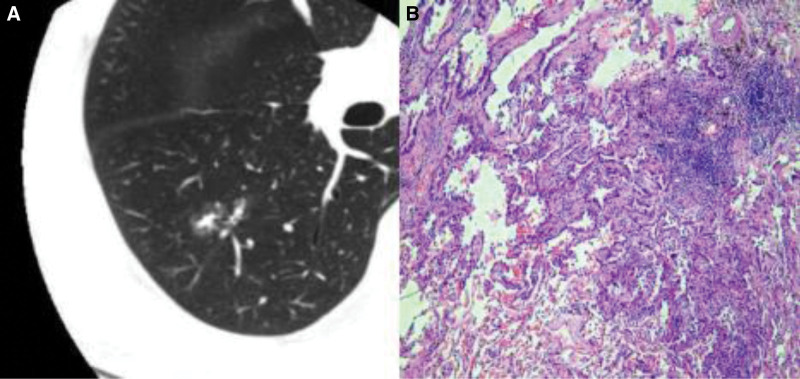
(A) The CT image and (B) the pathological image.

**Figure 3.**
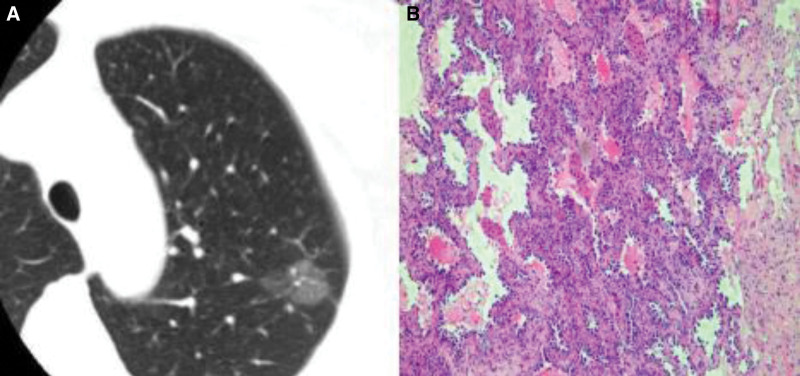
(A) The CT image and (B) the pathological image.

Figures 1–3 show CT images and corresponding pathological findings of 3 patients with different types of lung adenocarcinoma.

### 3.4. Correlation analysis of CT features and differentiation degree in MLA patients

Spearman correlation analysis showed that the long diameter of MLA lesions and the incidence rates of puncture sign, lobular sign, cavity sign, vascular cluster sign and pleural depression sign were negatively correlated with the degree of differentiation *(r* = −0.311, −0.361, −0.378, −0.307, −0.239, −0.242, all *P* < .05). The occurrence rate of pGGN was positively correlated with the degree of differentiation (*R* = 0.127, *P* < .05), while the occurrence rate of circle or quasi-circle was not correlated with the degree of differentiation (*P* > .05), as shown in Table 3.

**Table 3:** Correlation analysis between CT signs and differentiation degree in MLA patients.

Indicators	*r*	*P*
Focal length to diameter	–0.311	<.05
pGGN	0.127	<.05
Circular or almost circular	0.012	>.05
Burr sign	–0.361	<.05
Lobulation	–0.378	<.05
Cavitation character	–0.307	<.05
Vascular cluster sign	–0.239	<.05
Pleural depression sign	–0.242	<.05

GGNs = ground-glass nodules, MLA = multifocal lung adenocarcinoma.

### 3.5. Relationship between CT features and lymph node metastasis in MLA patients with different metastases

Spearman correlation analysis showed that the long diameter, round or round shape, lobular sign and vascular cluster sign of MLA patients were correlated with lymph node metastasis (*R* = 0.415, −0.367, 0.313, 0.329, all *P* < .05). There was no correlation between the occurrence rates of pGGN, burr sign, cavity sign, and pleural depression sign and lymph node metastasis (all *P* > .05), as shown in Table 4.

**Table 4:** Relationship between CT features and lymph node metastasis in MLA patients with different metastases.

Indicators	*r*	*P*
Focal length to diameter	0.415	<.05
pGGN	0.012	>.05
Circular or almost circular	–0.367	<.05
Burr sign	0.031	>.05
Lobulation	0.313	<.05
Cavitation character	0.028	>.05
Vascular cluster sign	0.329	<.05
Pleural depression sign	0.014	>.05

GGNs = ground-glass nodules, MLA = multifocal lung adenocarcinoma.

## 4. Discussion

Lung adenocarcinoma can be divided into atypical adenomatous hyperplasia, adenocarcinoma in situ, microinvasive adenocarcinoma, and invasive adenocarcinoma according to the presence and degree of surrounding invasion of cancer cells.^[7]^ With the development of imaging technology and the enhancement of people’s awareness of physical examination, chest CT physical examination is becoming more and more common, and the detection rate of pulmonary nodules is also significantly higher.^[8]^ Although most lung nodules are benign, some of them are early manifestations of lung cancer, so it is of great significance to scientifically and accurately evaluate lung nodules.^[9]^ This study provides a comprehensive analysis of the correlation between CT features and clinicopathological characteristics in patients with MLA presenting as GGNs. While previous studies have primarily focused on solitary GGNs, our research addresses the critical gap in understanding the imaging manifestations of MLA, a more complex and clinically challenging entity. By systematically comparing CT findings across preinvasive, microinvasive, and invasive adenocarcinoma groups, this study offers valuable insights into the diagnostic and prognostic potential of CT imaging in MLA.

Previous studies mainly focused on isolated GGNs, while there were few related reports on the imaging manifestations of MLA.^[10]^ In this study, 80 patients with MLA confirmed by pathology in our hospital were studied, and the correlation between CT signs and clinicopathological features of MLA patients was analyzed. According to the results of this study, there was no statistical difference in the lesion location between the preinvasive lesion group, microinvasive adenocarcinoma group and invasive adenocarcinoma group, so it is not possible to distinguish the pathological type of MLA simply based on the lesion location. The growth of cancer cells adherent to the alveolar wall increases the local density and forms ground-glass density. In the absence of alveolar cavity collapse and surrounding infiltration, CT shows pure GGNs; in the presence of alveolar cavity collapse, surrounding tissue infiltration and fibrous tissue hyperplasia, CT shows partial solid nodules.^[11–13]^ The results of this study showed that there were statistically significant differences in the types of GGNs between the preinvasive lesion group, the microinvasive adenocarcinoma group and the invasive adenocarcinoma group, and there were statistically significant differences between the pairwise comparisons, which confirmed the above views. With the growth of cancer cells, part of the alveolar structure is destroyed, and the punctate low-density transparent shadow within 5 mm can be seen in the undestroyed part of CT images, which is the vacuolar sign.^[14–18]^ At the same time, due to the difference of tumor growth and differentiation speed and invasion degree, the edge of the lesion is burred and lobulated, which are respectively burr sign and lobulated sign.^[19]^ The density of atypical adenomatous hyperplasia, adenocarcinoma in situ and microinvasive adenocarcinoma is relatively low, the contrast with the surrounding tissue is small, and the growth and invasion rate is relatively uniform in all directions, so the appearance is usually round or oval with blurred boundary. The invasive adenocarcinoma has a larger invasion area, higher density of lesions, and uneven growth and invasion. Air bronchial signs can be seen in the lesions, and the morphology is mostly irregular with relatively clear boundary. The collapse of the alveolar wall, fibrosis and contraction caused by the invasive growth of the tumor, and the vascular cluster and pleural depression sign can be seen on CT images.^[20–22]^ Therefore, irregular shape, clear boundary, vacuole sign, burr sign, lobulation sign, vascular cluster sign, air bronchial sign, pleural depression sign can be regarded as the typical malignant signs of MLA. The results of this study showed that there were statistically significant differences in nodule morphology, lobulation sign, vacuolation sign, burr sign, air bronchial sign, pleural depression sign, tumor–lung interface, and vascular characteristics among the preinvasive lesion group, microinvasive adenocarcinoma group, and invasive adenocarcinoma group, which were consistent with the above analysis.

On the other hand, the results of this study showed that there were significant differences in the lesion diameter, round or quasi-round, lobulated sign, and vascular cluster sign between the metastatic subgroup and the nonmetastatic subgroup, suggesting that lymph node metastasis may be affected by the above indicators. By the spearman correlation analysis, patients with MLA focal length to diameter and round or class round occurrence rate, rate, blood vessels, cluster Ye Zheng has occurrence rate associated with lymph node metastasis, suggested that when lesions in patients with large and round or class round, Ye Zheng, blood vessels, cluster prompt lymph node metastasis is likely to rise, the reason may be that: Circular or quasi-circular appearance indicates that the tumor growth rate is more balanced and not easy to metastasize. The pathological basis of lobulation sign is multinucleation and uneven growth rate of tumor, which is related to metastasis. MLA initially appears as ground glass nodules, which can develop into some substantial component nodules.^[23,24]^ The solid components of preinvasive lesions and microinvasive adenocarcinoma are usually presented as punctured density increasing shadows, while the invasive adenocarcinoma is usually presented as lumps and aggregates, confirming that the solid components of MLA are gradually increasing in the pathological development process from preinvasive lesions, microinvasive adenocarcinoma to invasive adenocarcinoma.^[25]^ At present, there is no unified standard for quantitative measurement and analysis of GGNs by CT. Many studies have shown that the larger the nodules and solid nodules are, the more likely they are to be invasive adenocarcinoma.^[26–30]^ In this study, the quantitative CT uptake numbers of MLA patients with different pathological types were compared and it was found that the average size of nodules and the average size of solid nodules were statistically significant between the preinvasive lesion group, the microinvasive adenocarcinoma group and the invasive adenocarcinoma group. Further pairwise comparison showed that the invasive adenocarcinoma group was larger than the other 2 groups. There was no significant difference between the preinvasive lesion group and the microinvasive adenocarcinoma group, suggesting that the invasive adenocarcinoma group can be identified to a certain extent by measuring the overall size of nodules and the size of solid nodules, while the differentiation between the preinvasive lesion group and microinvasive adenocarcinoma group needs further study.

This study has several limitations that should be acknowledged. First, the sample size of 80 patients, although sufficient for preliminary analysis, may still be relatively small for generalizing the findings to a broader population. Future studies with larger cohorts are needed to validate our results and enhance their statistical power. Second, the study population was recruited from a single center, which may introduce regional and institutional biases. The prevalence of MLA and the distribution of clinicopathological characteristics may vary across different geographic regions and ethnic groups. Therefore, the applicability of our findings to other populations, such as those in Western countries or regions with different healthcare systems, remains to be verified. Multi-center studies involving diverse patient populations are recommended to address this limitation. Third, the study relied on CT imaging data obtained from a specific type of equipment (GE Light Speed VCT 64-slice spiral CT). Although standardized protocols were followed, differences in imaging equipment, such as resolution, slice thickness, and reconstruction algorithms, may affect the interpretation of CT features. For instance, higher-resolution scanners might detect subtle signs, such as vacuolation or vascular changes, more accurately, potentially influencing the results. Future studies should consider incorporating data from multiple imaging platforms to assess the robustness of the findings across different equipment types. Finally, the retrospective design of the study inherently limits the ability to control for confounding factors. Variations in patient preparation, scanning parameters, and radiologist interpretation could introduce bias. Prospective studies with standardized protocols and blinded assessments are needed to further validate our conclusions. Despite these limitations, our findings contribute to the growing body of evidence on the imaging-based evaluation of MLA. By identifying specific CT features associated with pathological subtypes and lymph node metastasis, this study provides a foundation for future research and clinical practice. However, caution should be exercised when applying these findings to other populations or settings, and further validation is necessary to ensure their generalizability.

In conclusion, CT signs of MLA patients are closely related to pathological features, which can provide a certain basis for clinical diagnosis and pathological classification. However, due to the limited sample size, case selection may be biased, and there are many observation indicators, and multivariate regression analysis is not carried out, which needs to be further improved in the future.

## Author contributions

**Conceptualization:** Meng Zhou, Chunlin Li.

**Data curation:** Meng Zhou, Jiuchen Wang, Huadong Chen, Chunlin Li.

**Formal analysis:** Meng Zhou, Jiuchen Wang, Huadong Chen, Chunlin Li.

**Investigation:** Meng Zhou, Jiuchen Wang, Huadong Chen, Chunlin Li.

**Methodology:** Meng Zhou, Jiuchen Wang, Weihua Tang, Chunlin Li.

**Supervision:** Meng Zhou, Jiuchen Wang, Weihua Tang, Chunlin Li.

**Writing – original draft:** Meng Zhou, Weihua Tang, Chunlin Li.

**Writing – review & editing:** Meng Zhou, Chunlin Li
